# Endogenous listeria monocytogenes endophthalmitis in a cirrhotic patient

**DOI:** 10.1002/ccr3.7670

**Published:** 2023-07-06

**Authors:** Huping Song, Yunyun Zhou, Tao Chen, Jingbo Wang

**Affiliations:** ^1^ Department of Ophthalmology Shaanxi Eye Hospital, Xi'an People's Hospital (Xi'an Fourth Hospital) Xi'an China

**Keywords:** case report, endogenous endophthalmitis, listeria monocytogenes, pigmented hypopyon, rare, serious

## Abstract

Endogenous endophthalmitis caused by listeria monocytogenes is rare and serious, which is easily misdiagnosed initially. We present one case of endogenous endophthalmitis due to listeria monocytogenes in a cirrhotic patient, whose diagnosis was delayed 17 days after admission.

## 1 INTRODUCTION

Most cases of endophthalmitis are caused by exogenous pathogens, only 5% ~ 10% are of endogenous origin,^1^ among which Listeria monocytogenes is a rare and serious one. We presented one case of endogenous endophthalmitis due to Listeria monocytogenes in a cirrhotic patient.

## 2 CASE REPORT

A 72‐year‐old man presented with foreign body sensation for 1 month, redness and decreased vision in the right eye for 10 days. Ten days before being referred to our hospital, he was diagnosed uveitis and secondary glaucoma in the right eye, and received ocular medicine treatment of anti‐inflammatory and lowering intraocular pressure, but the symptoms were not significantly relieved. His personal history includes liver cirrhosis complicated with hypoalbuminemia due to Hepatitis B, and all have not been treated regularly. He underwent splenectomy for hypersplenism and underwent transjugular intrahepatic portosystemic shunt for liver cirrhosis complicated with esophageal varices hemorrhage. He regularly took antihypertensive drugs due to hypertension.

**FIGURE 1:**
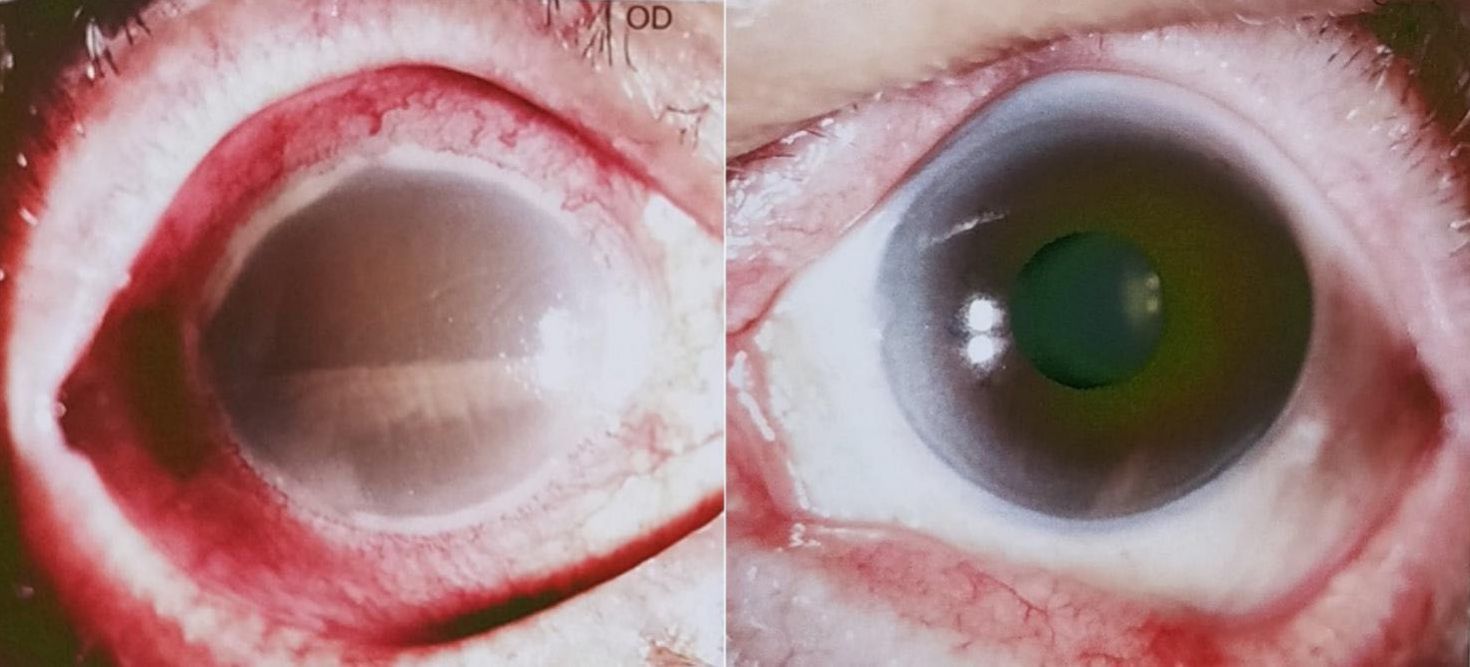
Slit lamp microscope examination images showing the lower 2/3 of the anterior chamber filling with tan pigmented hypopyon in the right eye.

**FIGURE 2:**
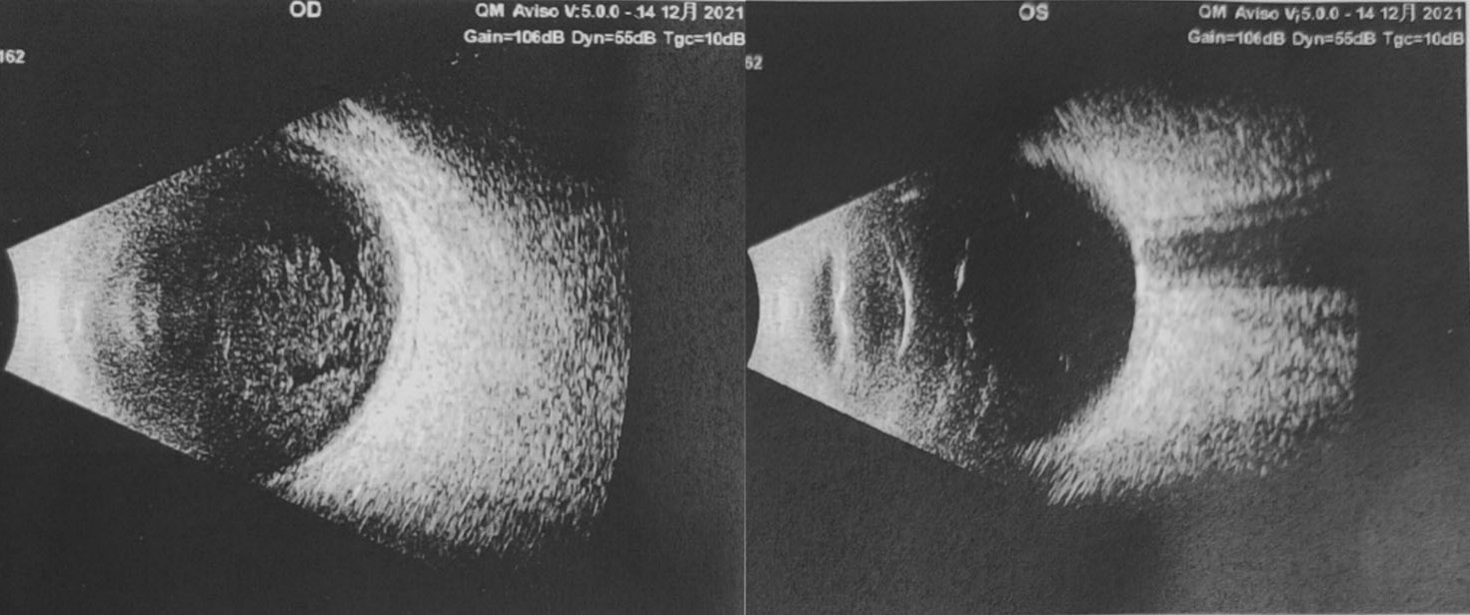
B‐scan ultrasonography revealing vitreous opacity and vitreous infiltration at the posterior hyaloid membrane, no retinal detachment in the right eye.

On initial ophthalmological examination, visual acuity (VA) was light perception (LP) in the right eye and 20/50 in the left eye, the intraocular pressure (IOP) was 22.6 mmHg in the right eye and 11.9 mmHg in the left eye. The examination of the right eye revealed conjunctival hyperemia and edema, and corneal edema, pigmented keratic precipitates (KPs) 3+, and the lower 2/3 of the anterior chamber is filled with pigmented hypopyon (Figure 1). Therefore, neither the lens nor the fundus could be clearly visualized. An ultrasound scan of the eye revealed vitreous opacity and vitreous infiltration at the posterior hyaloid membrane (Figure 2). The left eye examination was unremarkable. His total protein content in the blood reduced to 46.0 g/L (range 64.0 ~ 83.0 g/L), and the albumin content reduced to 21.0 g/L (range 35.0 ~ 52.0 g/L). The total bilirubin content in the blood increased to 28.9 μmol/L (range 3.4 ~ 20.5 μmol/L), and the direct bilirubin content increased to 10.2 μmol/L (range 0.0 ~ 8.6 μmol/L). Chest computed tomography scanning revealed bilateral pleural thickening and bilateral pleural effusion.

The initial diagnosis was phacolytic endophthalmitis, secondary glaucoma and cataract of the right eye, considering pigmented fluid in the anterior chamber and no previous cataract surgery. Treatments with vitrectomy, phacoemulsification, anterior chamber irrigation, and intraocular injection of vancomycin were provided emergently. During the operation, slightly opacified lens, a large amount of brown sand‐like opacity in the anterior chamber and vitreous cavity were detected. The sample of vitreous humor was taken for microbiological examination.

On the next day after surgery, VA increased to counting figures (CF)/10 cm, and IOP recovered to 16.4 mmHg, ocular examination revealed corneal edema with endothelial folds, clear anterior chamber, and fundus could not be visualized clearly. On the second day after surgery, VA decreased to CF/5 cm, and IOP increased to 25.8 mmHg, ocular examination showed slightly relieved corneal edema with endothelial folds, and 2+ flare in the anterior chamber. Local IOP‐lowering agents were used. On the sixth day post‐operation, VA continued to be CF/5 cm and IOP was 24 mmHg, while tan opacity of the anterior chamber increased gradually, and fundus still could not be visualized. An anterior chamber irrigation was performed urgently to flush out the tan particles, and clear vitreous media was observed; then, a small amount of triamcinolone was injected to the anterior chamber. The microbiological study of the vitreous humor was negative. One day after the irrigation (Day 8), VA increased to hand movement (HM)/30 cm, and IOP was 18.3 mmHg, pigmented KPs 2+ and 2+ flare was observed in the anterior chamber. The ocular fundus could not be visualized. Four days after the irrigation (Day 11), the patient complained with right eye pain, slit‐lamp examination showed obvious corneal edema, a 1‐mm hypopyon and 3+ flare. Intravenous vancomycin and ceftazidime were infused, and subconjunctival ceftazidime were injected immediately for suspected infectious endophthalmitis. A diagnostic and therapeutic vitreous irrigation was performed on the next day with subsequent administration of empirical intravitreal vancomycin. During the operation, blurred boundary of optic disk was visualized, dissolved peripheral retina was scattered with patchy preretinal hemorrhage, the peripheral retina was bluish‐gray bulge and membranous proliferation was seen on the detached retina. Therefore, laser coagulation and silicone oil tamponade were performed. Anterior aqueous humor samples obtained were sent for culture, which was cultured on aerobic and anaerobic blood agar. Vancomycin and ceftazidime were administered intravenously for 5 days post‐surgery. Three days after the surgery (Day 15), the VA was LP and IOP was 14.7 mmHg, gray‐white corneal edema and endothelial folds were visible, and KPs 2+ in the right eye. Five days after vitreous irrigation (Day 17), culture of the anterior aqueous humor grew Listeria monocytogenes. Due to the COVID‐19 epidemic, the patient did not follow up. Fifteen days later (Day 30), he presented with fever to the emergency department. By the hand‐held slit lamp biomicroscope, no obvious inflammation was observed in his right eye. The following Day 3 (Day 33), he died in the intensive care unit due to severe pneumonia.

## 3 DISCUSSION

Besides the blood‐borne seeding, endogenous endophthalmitis is related to the presence of predisposing factors, such as cirrhosis.^2^ Listeria monocytogenes is a ubiquitous Gram‐positive rod, which rarely produces invasive disease that primarily affects immunosuppressed patients, chronic diseases, malignancies, the elderly and pregnant women; mainly causing bacteremia and meningitis.^2^, ^3^, ^4^ Although epidemic foodborne and vertical transmission are established routes of infection, most cases are sporadic, so the origin and mode of transmission of the organism are unknown. Listeria monocytogenes endophthalmitis is exceedingly rare, which accounts for less than 4% of cases of bacterial endophthalmitis,^5^, ^6^ and it was first reported in the literature in 1967.^7^ Ocular symptoms of Listeria endophthalmitis include pain (87%), eye redness (65%), decreased vision (56%), and photophobia (13%), and the clinical features include elevated IOP (93%), fibrinous anterior chamber reaction (67%), and a dark hypopyon (85%).^1^

Despite taking intraocular humor samples for microbiological test, it is common not to find the source of the infection in endogenous endophthalmitis. The cause of bacterial endophthalmitis was isolated by recovering Listeria monocytogenes from the anterior chamber (86%), vitreous humor (78%), and blood culture (23%).^1^, ^8^, ^9^, ^10^ The direct performed Gram stain from ocular samples was positive in merely 40% cases; thus, the importance of microbiology culture was emphasized owing to the higher sensitivity.^1^ In this case, the diagnosis was delayed 17 days after admission. The time period between the presentation of the patient and the beginning of an effective treatment widely ranges from 4 to 32 days, which mainly depends on selected samples, the bacterial load in the sample, prior administered anti‐infective drugs, and techniques for pathogens identification.^1^

Currently, there is no standard treatment protocols. A quarter of patients had five or more antibiotics and more than half received antibiotics via three or more routes, which may be owing to the concerns about ocular drug penetration and the limited evidence available to guide selection of antibiotics.^5^ Most of patients who present Listeria endophthalmitis have poor visual acuity results even with adequate antibiotic treatment, especially due to a delay in the diagnosis since it is a rare pathology.^3^ It is reported that from 2011 to 2016 in 19 provinces of China, the overall case‐fatality rate of invasive listeriosis was 25.7%.^11^ The most common clinical manifestation of non‐perinatal listeriosis cases was septicemia, central nervous system infection and pneumonia.^11^ Although we cannot determine whether this patient died of invasive listeriosis, we still need to be aware that listeria endophthalmitis may also carries a worse systemic outcome.

In conclusion, listeria endophthalmitis is rare, however which can cause poor visual prognosis, even severe systemic outcome. While treating patients with a massive fibrinous exudate with a tan to tan‐black pigmented anterior chamber abscess, we should recall the potential differential diagnosis. Diagnosis of listeria endophthalmitis is made only after culture, which can delay proper antibiotic coverage. The importance of taking intraocular fluid for microbiological culture for the diagnosis and early treatment as soon as possible is highlighted.

## AUTHOR CONTRIBUTIONS

**Huping Song:** Conceptualization; supervision; writing – review and editing. **Yunyun Zhou:** Data curation; writing – original draft. **Tao Chen:** Investigation. **Jingbo Wang:** Writing – original draft.

## FUNDING INFORMATION

This study did not receive any funding in any form.

## CONFLICT OF INTEREST STATEMENT

The authors declare that they have no competing interests.

## CONSENT

Written informed consent was obtained from the patient's next of kin to publish this report in accordance with the journal's patient consent policy.

## Data Availability

The datasets used and/or analyzed during the current study are available from the corresponding author upon reasonable request.
